# Maintaining memory of silencing at imprinted differentially methylated regions

**DOI:** 10.1007/s00018-016-2157-6

**Published:** 2016-02-16

**Authors:** Hsiao P. J. Voon, Richard J. Gibbons

**Affiliations:** Department of Biochemistry and Molecular Biology, Monash University, Clayton, VIC 3800 Australia; University of Oxford, Oxford, UK; MRC Molecular Haematology Unit, Weatherall Institute of Molecular Medicine, John Radcliffe Hospital, Headington, Oxford, OX3 9DS UK

**Keywords:** Genomic imprinting, Heterochromatin, PGC7, Zfp57, ATRX, Daxx, H3.3

## Abstract

Imprinted genes are an exceptional cluster of genes which are expressed in a parent-of-origin dependent fashion. This allele-specific expression is dependent on differential DNA methylation which is established in the parental germlines in a sex-specific manner. The DNA methylation imprint is accompanied by heterochromatin modifications which must be continuously maintained through development. This review summarises the factors which are important for protecting the epigenetic modifications at imprinted differentially methylated regions (DMRs), including PGC7, ZFP57 and the ATRX/Daxx/H3.3 complex. We discuss how these factors maintain heterochromatin silencing, not only at imprinted DMRs, but also other heterochromatic regions in the genome.

## Introduction

**Fig. 1:**
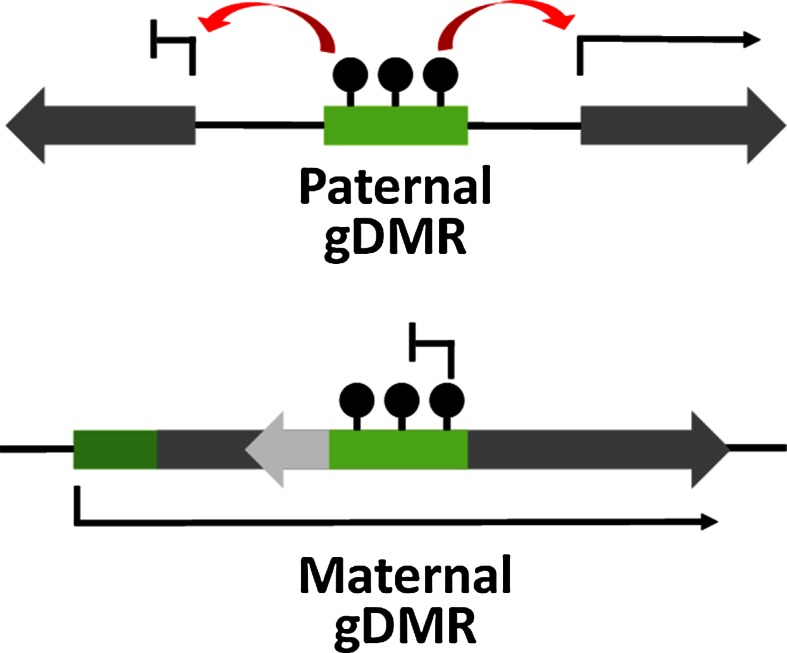
Paternal and maternal methylated gDMRs are associated with distinct genomic locations. Paternally methylated gDMRs are located at intergenic regions and methylation at gDMRs specifies allele-specific expression of genes at flanking genes. Maternally methylated gDMRs are located at intragenic sites and methylation can directly repress transcription of an antisense transcript as well as influence the expression of flanking genes within the cluster

Imprinted genes are a specialised group of genes in mammalian genomes which are monoallelically expressed in a parent-of-origin dependent manner. Approximately 100–150 imprinted genes have been identified in both the mouse and the human genome to date [1]. The majority of these genes are arranged in chromosomal clusters and imprinted monoallelic expression of multiple genes in a cluster can be controlled by a single germline differentially methylated region (gDMR) within the cluster [2–5]. The differential methylation at gDMRs is established in parental germlines and maintained through development to facilitate imprinted gene expression. Of the 20 gDMRs identified to date, only three gDMRs acquire DNA methylation in the paternal germline while the remainder are methylated on the maternally inherited allele [6]. The maternal and paternal gDMRs are associated with distinct genomic locations; the three paternally methylated gDMRs are located at intergenic sites while maternally methylated gDMRs are CpG islands (CGIs) which are intragenic and embedded within transcriptional units [6] (Fig. 1). The different positions of maternal vs paternal gDMRs possibly arises from sex-specific differences in germ cell development including differences in gene expression, timing of methylation and rounds of cell division.

In addition to differential DNA methylation, gDMRs are also associated with differential histone modifications in accordance with the methylation status [7–10]. The unmethylated allele is enriched for modifications associated with active chromatin such as H3K4me3 [8, 10–12], which also protects against de novo DNA methylation. Conversely, the methylated allele is enriched for modifications associated with constitutive heterochromatin such as H3K9me2/3 [8, 10, 12]. These modified histones are present in both sperm and oocytes and contribute to the maintenance of imprinting through development.

**Fig. 2:**
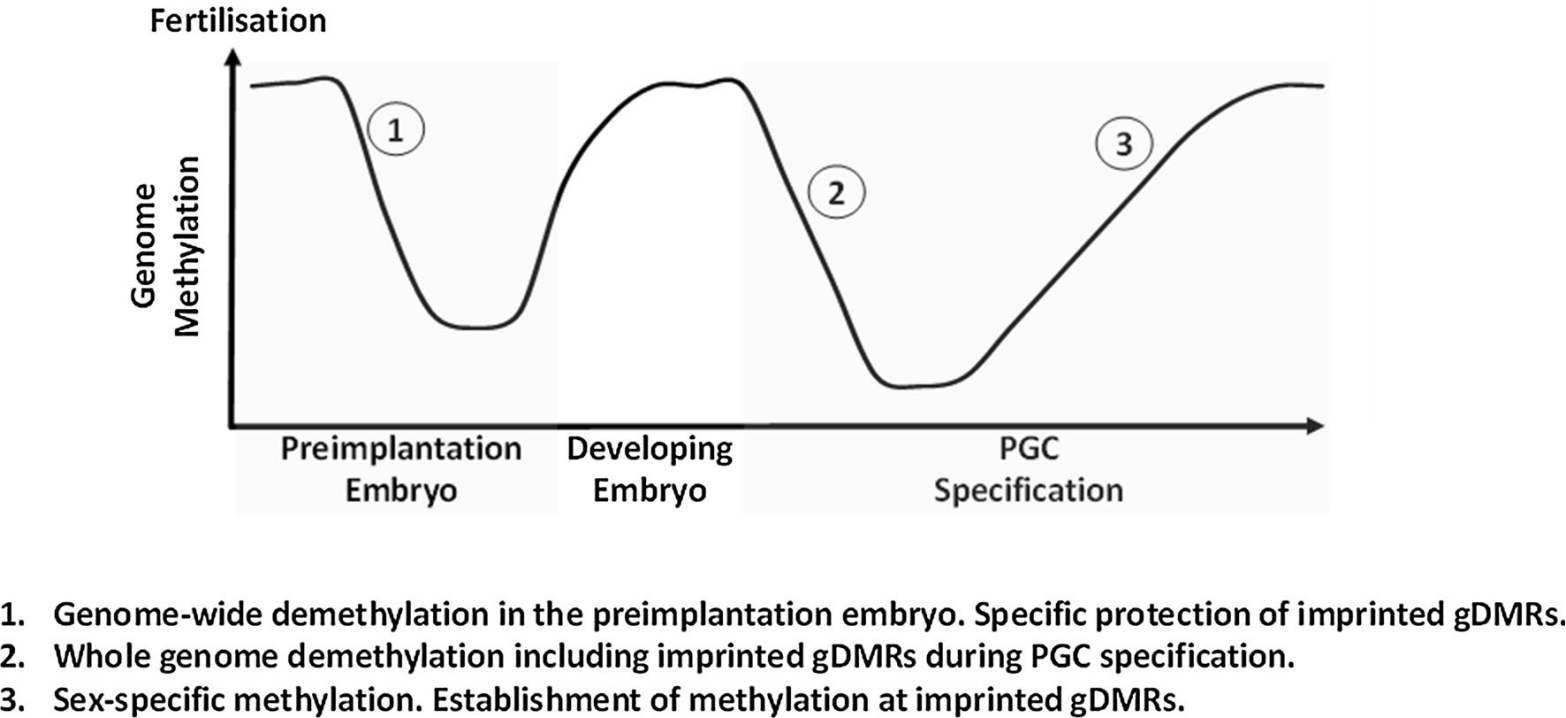
Cycle of methylation through mammalian development. Mammalian cells undergo two major waves of demethylation. The first wave occurs during the pre-implantation period when most methylation, including most sex-specific methylation is cleared, with the exception of the imprinted gDMRs. A second wave of demethylation occurs in PGCs when all methylation is cleared, including gDMRs, except for certain repeats. The genome is then re-methylated in a sex specific manner and parental specific methylation at gDMRs is established

To correctly specify imprinted gene expression in the developing organisms, DNA methylation at gDMRs must survive a wave of demethylation which occurs during very early development [13] (Fig. 2). Mammalian genomes undergo two cyclical waves of demethylation during development. The first wave of demethylation occurs post-fertilisation in the pre-implantation embryo. The paternal genome is subjected to active demethylation during this period while the maternal genome undergoes passive demethylation [14]. The imprinted gDMRs and certain repeats are protected from this indiscriminate demethylation, and DNA methylation across the genome is subsequently restored during development. The second wave of demethylation occurs during germline specification in primordial germ cells when the entire genome (including gDMRs) is demethylated with the exception of IAP retrotransposon repeats in the mouse [15, 16]. This resets the genome and methylation at gDMRs is then re-established in a sex-specific manner in the maturing germ cells [17, 18]. It is worth nothing that differential methylation at this stage is not limited to gDMRs but is instead prevalent across the genome [17, 18]. Much of the differential methylation acquired in the germline is subsequently lost during the wave of preimplantation demethylation, with the exception of imprinted DMRs and certain repeats (e.g. IAPs) [17, 18]. Protection of DNA methylation at gDMRs is critical during the pre-implantation period and two proteins, PGC7 and Zfp57, are known to be important for this process.

## PGC7 protects imprinted methylated DMRs from passive demethylation

PGC7 (also known as Stella or Dppa3) was first identified as a protein which is highly expressed in primordial germ cells (PGCs) [19]. Expression of PGC7 is maintained through to oocyte maturation [19] and maternally provided PGC7 persists into the pre-implantation embryo where PGC7 localises to the pronuclei [20]. Zygotes derived from PGC7 KO oocytes failed to develop into blastocysts, indicating that PGC7 is critical during the post-fertilisation period [20]. This coincides with the timing of genome-wide demethylation and, combined with the genome-specific localisation of PGC7, suggested a role for PGC7 in controlling DNA methylation. In agreement with this, zygotic genomes derived from PGC7 KO oocytes had reduced levels of DNA methylation, with a particularly pronounced effect at the maternally-derived genome [20]. Loss of methylation was observed at a number of maternally methylated DMRs (Peg1, Peg3 and Peg10) as well as two paternally methylated DMRs (Rasgrf1 and H19) and IAP repeats [20].

The specificity of this altered methylation was found to be conferred by the preferential localisation of PGC7 to H3K9me2 modified chromatin [21]. The two parental genomes are not equivalent at fertilisation; the maternal genome is fully chromatinised and enriched for H3K9me2 while the paternal genome is subjected to extensive remodelling [22]. The bulk of the genome in mature sperm is packaged around protamines and histones are retained at only a few exceptional regions (1 % in mouse, 10 % in human remain associated with histones) [23, 24] including the imprinted DMRs [21]. During the post-fertilisation period, protamines are replaced with histones and the paternal-derived genome is subjected to active demethylation [14] via Tet3-mediated conversion of 5-methylcytosine (5mC) to 5-hydroxymethylcytosine (5hmC) [25–27]. The paternally methylated DMRs and the maternal-derived genome are largely protected from active demethylation and PGC7 plays an important role in this process [21].

The preferential binding of PGC7 to the maternal-derived genome suggested that the H3K9me2 modification may be important for PGC7 localisation. Consistent with this, PGC7 was found to interact most strongly with H3K9me2 modified histone peptides in in vitro binding assays and a pull-down of endogenous PGC7 demonstrated an enrichment for H3K9me2 [21]. Furthermore, both knockdown of G9a (a lysine methyltransferase which catalyses H3K9me2) and forced expression of Jhmd2a (an H3K9me2 specific demethylase) was found to abrogate the preferential localisation of PGC7 to the maternal genome [21]. Lastly, the two paternal DMRs (Rasgrf1 and H19) which were hypomethylated in PGC7 KO embryos were enriched for H3K9me2 in mature sperm [21], suggesting that H3K9me2 mediated binding of PGC7 protects these regions against active demethylation during the post-fertilisation period.

**Fig. 3:**
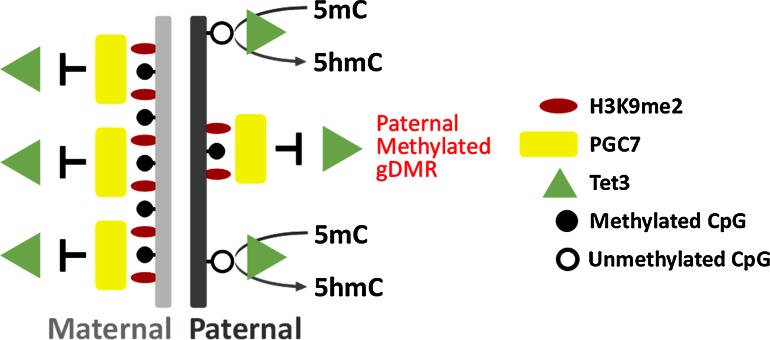
PCG7 binding is dependent on H3K9me2 and protects against Tet3-mediated active demethylation. PGC7 localises to the maternal genome and paternally methylated gDMRs in pre-implantation embryos. Localisation of PGC7 is dependent on H3K9me2 which coats the maternal genome but is largely absent on the paternal genome with the exception of methylated gDMRs. Binding of PGC7 protects the maternal genome and paternally methylated gDMRs from Tet3-mediated demethylation

Forced expression of Jhmd2a resulted in strong binding of Tet3 to both maternal and paternal derived genomes and knockout of PGC7 resulted in abnormal Tet3 mediated conversion of 5mC to 5hmC on the maternal-derived genome. Furthermore, recombinant PGC7 was found to directly repress the enzymatic activity of Tet3 in in vitro assays [21]. Combined, these results suggest that the H3K9me2 modification attracts PGC7 to inhibit Tet3 demethylation during early embryonic development. This appears to be a general process which is important for preserving the integrity of the maternal genome but is also important for maintaining the DNA methylation of a number of maternal and paternal DMRs (Fig. 3).

## Zfp57 recruits the KAP1 co-repressor to methylated DMRs

In addition to PGC7 a second protein, Zfp57, is known to play an important role in maintaining DNA methylation at imprinted DMRs in early embryos. Zfp57 is one of hundreds of KRAB-ZFPs which are present in mammalian genomes [28]. Each KRAB-ZFP is comprised of a zinc finger domain which binds DNA, often in a sequence specific manner, and a Kruppel Associated Box (KRAB) domain which recruits the KAP1 (aka Trim28) co-repressor complex [28]. KAP1 is a scaffold protein which interacts with a number of heterochromatin proteins including DNMTs [29], Setdb1 (aka ESET) and Heterochromatin Protein 1 (HP1) [30]. The KRAB-ZFP mediated targeting of KAP1 to specific genomic regions, is therefore able to induce heterochromatin formation and transcriptional silencing. At imprinted DMRs, the KAP1 co-repressor complex is recruited by Zfp57 and this interaction is important for maintaining methylation through the pre-implantation period. The role of Zfp57 in imprinting came to light when mutations in Zfp57 were identified in cases of transient neonatal diabetes; this was associated with hypomethylation of multiple imprinted loci [31]. Zfp57 is highly expressed in ES cells and downregulated upon differentiation [32]. In adult tissues, expression of Zfp57 is primarily restricted to testes and ovaries, reflecting its critical role in embryogenesis [33]. Maternal-zygotic loss of Zfp57 resulted in no live births, while zygotic KO of Zfp57 resulted in partial lethality [33]. This demonstrated that maternally provided Zfp57 was able to partly compensate for loss of zygotic Zfp57 and indicates that Zfp57 is critical during early embryogenesis.

ChIP-seq revealed that Zfp57 was bound to all known imprinted gDMRs and this binding was specific to the methylated allele [34]. In silico analyses identified a TGCCGC/(N) [29, 34] motif under Zfp57 binding sites and this was confirmed in gel-shift assays performed with the zinc finger domain of Zfp57 [29]. This fragment of Zfp57 was found to bind specifically to the TGCCGC sequence with a particular preference for the methylated motif [29]. The heptamer motif is found at all mouse gDMRs as well as some human gDMRs [29] and may contribute to the selective targeting of these regions for protection against demethylation. Knockout of Zfp57 in embryos or ES cells resulted in loss of methylation at a number of imprinted gDMRs [29, 33, 35] and re-expression of exogenous Zfp57 in null ES cells was unable to restore methylation [35]. This suggests that the continued presence of Zfp57 is required for maintaining methylation at gDMRs in mouse ES cells.

**Fig. 4:**
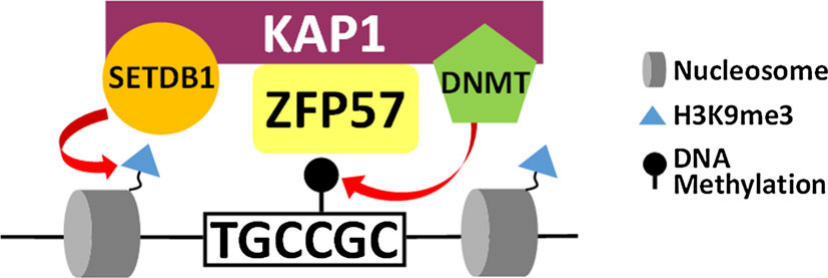
Zfp57 recruits the KAP1 co-repressor complex to maintain silent modifications. Zfp57 binds preferentially to a methylated motif (TGCCGC/N) found at imprinted gDMRs. Zfp57 recruits the KAP1 scaffold protein which interacts with both DNMT and Setdb1, which catalyse DNA methylation and H3K9me3 respectively

This process is dependent on Zfp57 recruitment of KAP1 to methylated gDMRs as expressing a KAP1-interaction defective, KRAB-domain deleted Zfp57, is unable to rescue the knockout [35]. KAP1 is a scaffold protein which is able to induce de novo DNA methylation through the recruitment of multiple heterochromatin proteins. KAP1 interacts with both de novo (DNMT3a/b) and maintenance (DNMT1) methyltransferases [29] and targeting KAP1 to specific genomic sites is sufficient to induce DNA methylation [36]. In addition, KAP1 is also able to recruit heterochromatin protein 1 (HP1) and Setdb1, a lysine methyltransferase which catalyses H3K9me3 [30]. Given the strong association between H3K9me3 and DNA methylation, it is likely that the KAP1/Setdb1/H3K9me3 axis is also important for the maintenance of DNA methylation at these regions. The Zfp57 mediated recruitment of the KAP1 co-repressor complex to methylated gDMRs is therefore able to attract heterochromatin proteins to maintain DNA methylation at these sites (Fig. 4).

## Chromatin modifications at imprinted DMRs

DNA methylation does not occur in isolation, but is instead inextricably linked to histone modifications. Three well characterised histone modifications—H3K4me3, H3K9me3 and H3K36me3—are particularly important in the context of genomic imprinting. H3K4me3 is a modification which is highly enriched around gene promoters and is permissive to transcription [37]. At imprinted DMRs, H3K4me3 is restricted to the unmethylated allele [8, 9] and the absence of H3K4me3 is a specific requirement for the establishment of DNA methylation at a number of maternally methylated gDMRs. Knockout of a H3K4-specific demethylase, LSD2, led to accumulation of H3K4me2/3 in oocytes and prevented the methylation of several maternal gDMRs [38]. This phenomenon can be explained by the ATRX-DNMT3-DNMT3L (ADD) domain found in DNMT3a/b and the DNMT3L co-factor, which guides the localisation of these methyltransferases. This ADD domain of DNMT3L has been demonstrated to bind to the histone H3 tail only when the H3K4 residue is unmethylated [39]. In DNMT3a/b, the ADD domain also interacts with the H3 tail and the catalytic activity of these methyltransferases is inhibited by H3K4me3 [40]. The localisation and activity of DNMT3a/b, and DNA methylation, is therefore dependent on the absence of H3K4me3.

In addition to the specific requirement for H3K4me0, the localisation of DNMT3a/b are also guided by interactions with HP1 [41, 42] which is in turn, dependent on H3K9me3 [43, 44]. The H3K9me3 modification is generally associated with heterochromatin and is particularly prominent at repetitive DNA such as the centromeric, pericentric and telomeric regions [45–47]. This modification is predominantly catalysed by the Suv39h methyltransferases at repeats [42, 47] and by Setdb1 at genic regions [48, 49], including the methylated DMRs [50]. The presence of H3K9me3 provides a binding site for HP1 [43, 44] which then recruits DNMT3a/b to catalyse DNA methylation [41, 42]. The H3K9me3 modification is therefore able to prime genomic regions for DNA methylation. The Zfp57 mediated recruitment of KAP1 to methylated gDMRs facilitates the maintenance of both H3K9me3 via Setdb1 and DNA methylation via interactions with DNMT3a/b, thereby ensuring the retention of DNA methylation at these sites.

In addition to the ADD domain, DNMT3a/b also harbours a PWWP domain which is able to interact with the H3K36me3 modification [51–53]. H3K36me3 is found within the bodies of active genes and is thought to prevent the aberrant initiation of transcription [54, 55]. The maternally methylated gDMRs are all CpG islands which are located within the body of a transcriptional unit and as such, are all enriched for H3K36me3 [9]. In addition to recruiting DNMTs, H3K36me3 also recruits the KDM5B H3K4me3-demethylase which protects these intragenic CGIs from aberrant histone modifications thereby facilitating the continued maintenance of silencing at these sites [56]. The double enrichment of H3K9me3 and H3K36me3 guide the binding of DNMT3a/b via the ADD and PWWD domains respectively, while H3K36me3 recruitment of KDM5B protects against the gain of H3K4me3 at the intragenic CGIs which comprise maternally methylated DMRs.

## Role of transcription and non-coding RNAs in imprinted gene expression

**Fig. 5:**
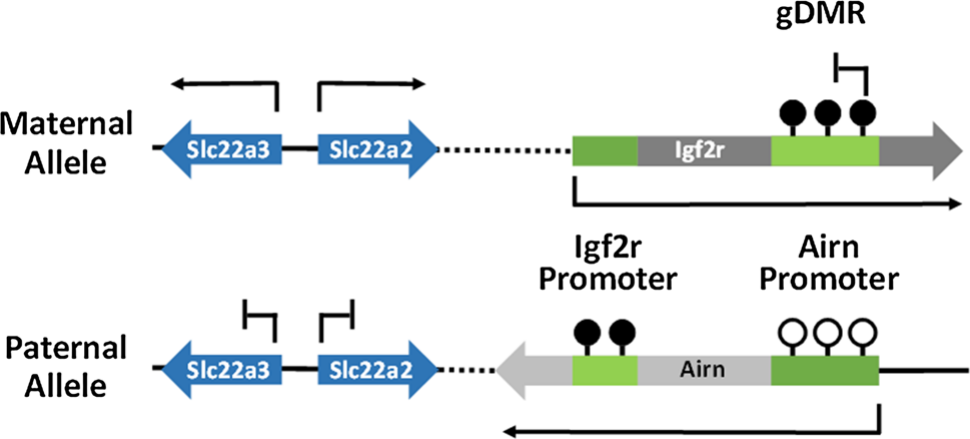
Example of non-coding RNA and transcription in imprinted gene expression. The Igf2r/Airn imprinted locus is an example locus which illustrates the role of transcription and non-coding RNA in controlling expression of imprinted genes. The maternal methylation at the gDMR prevents expression of the non-coding Airn transcript from the maternal allele. The gDMR is unmethylated at the paternal allele which allows expression of the Airn transcript. Transcription of Airn through the Igf2r promoter results in methylation at the paternal allele. The non-coding Airn transcript prevents expression of Slc22a2 and Slc22a3 from the paternal allele

The association between transcription and DNA methylation at imprinted gDMRs is further supported by studies on the corresponding non-imprinted allele. Many imprinted gene clusters express at least one long non-coding RNA (lncRNA) which is often arranged in the antisense orientation with respect to the methylated gDMR promoter (Fig. 5). Examples of this include the Igf2r/Airn [57], Gnas/Nespas [58], Snrpn/Snrpnlt [59], and the Kcnq1/Kcnq1ot1 loci [60]. At these regions, DNA methylation and silencing modifications suppresses the expression of the ncRNA from the maternal allele. However, the unmethylated paternal allele transcribes a lncRNA which has been associated with methylation and silencing of flanking genes in the imprinted cluster [61]. The exact mechanisms which drive this process are not well understood due to difficulties in separating the function of the ncRNA transcript from the process of transcription. Nonetheless, experiments where the ncRNA transcript was artificially truncated with the addition of a premature Poly-A signal or destabilising the transcript, have demonstrated that the ncRNA transcript is involved in specifying imprinted gene expression at some clusters [62, 63].

In addition, several other studies have indicated that the process of transcription may also play a role in this process [64, 65]. For example, at the Igf2r locus, transcription of the Airn ncRNA was demonstrated to be important for methylation and silencing of the Igf2r promoter on the paternal allele, independent of the Airn lncRNA [64]. In this study, a truncated Airn transcript was inserted in the reverse orientation so that transcription no longer traversed the Igf2r promoter. This arrangement preserved the expression of a truncated Airn transcript while abolishing transcription across the Igf2r promoter. The Igf2r promoter was normally methylated in a control line where the truncated Airn promoter was positioned in the forward orientation but methylation was lost when the Airn promoter was positioned in the reverse orientation [64]. This effectively demonstrates that both the lncRNA products and the process of transcription across promoters contributes to imprinted gene expression.

## ATRX/Daxx/H3.3 maintain H3K9me3 at methylated DMRs

The active transcription through the intragenic maternal methylated DMRs poses an additional challenge for the maintenance of methylation at these sites. The position of these imprinted DMRs means that these heterochromatic foci are subjected to transcription, and chromatin modifications must be continuously restored following the passage of RNA polymerase. This process has recently been demonstrated to be dependent on a histone variant, H3.3. Unlike the canonical histones (H3.1/H3.2), which are expressed and deposited only during S-phase, histone H3.3 is expressed throughout the cell cycle and replaces histones which are displaced by transcription [66, 67]. Consistent with this, ChIP-seq revealed that H3.3 predominantly localises to the promoters and gene bodies of active genes [68]. However, in mouse ES cells, H3.3 enrichment was also detected at a number of heterochromatic regions including telomeres, ERVs and imprinted DMRs [12, 68, 69]. Allele-specific ChIP-seq and ChIP-PCR revealed that H3.3 was preferentially enriched on the DNA methylated allele at all imprinted DMRs tested, suggesting a functional role for H3.3 in maintaining silencing at the methylated allele [12].

**Fig. 6:**
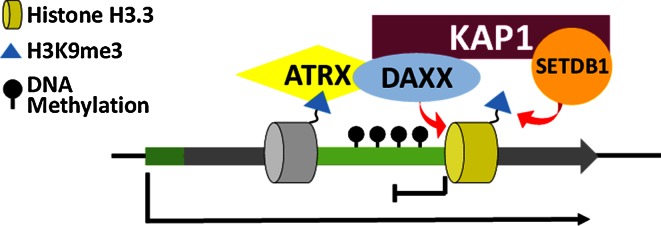
The ATRX/Daxx/H3.3 complex continuously maintain heterochromatin at imprinted DMRs. ATRX can directly bind H3K9me3 at imprinted DMRs to recruit Daxx and deposit the replication-independent H3.3 histone variant. Daxx interacts with the KAP1 co-repressor complex which recruits Setdb1 to catalyse H3K9me3 heterochromatin at these sites. The replication-independent nature of H3.3 ensures the continuous maintenance of heterochromatin at gDMRs throughout the cell cycle particularly during transcription

Two major chaperone complexes which deposit H3.3 have been identified. The HIRA complex is the major H3.3 chaperone at euchromatin genic sites [68, 70] while deposition of H3.3 at heterochromatin is dependent on the ATRX/Daxx complex [68, 71]. Daxx is the H3.3 chaperone in this complex [72–74] while ATRX is a chromatin remodeller, and both components are required for the incorporation of H3.3 at heterochromatin, including methylated DMRs. ChIP-seq revealed ATRX enrichment at a number of imprinted DMRs in mouse ES cells and allele-specific ChIP-PCR demonstrated preferential localisation to the methylated allele [12]. Similar to DNMT3, ATRX also harbours an ADD domain which specifically recognises H3K4me0 [75–77] but additionally it directly binds H3K9me3 [75–77]. ATRX also contains an HP1 binding motif (67). These interactions likely promote the specific localisation of ATRX/Daxx/H3.3 to the methylated DMR to facilitate allele-specific deposition of H3.3. Knockout of ATRX led to loss of H3.3 at imprinted DMRs which was co-incident with the loss of the H3K9me3 heterochromatin modification [12]. A similar phenomenon was observed at telomeres [78] and ERV repeats [69] where ChIP-reChIP assays demonstrated that H3.3 is modified with K9me3 (H3.3K9me3) [69, 78]. Furthermore, Daxx was shown to interact with KAP1 and Setdb1 was required for catalysing H3.3K9me3 at ERVs [69]. It is likely that an equivalent pathway operates at methylated DMRs where ATRX/Daxx deposits H3.3 which is modified to H3.3K9me3 by Setdb1. In this case, KAP1/Setdb1 could be recruited either by Zfp57 or Daxx, or a combination of both. The replication-independent nature of H3.3 would ensure continued maintenance of heterochromatin at methylated DMRs throughout the cell cycle and particularly when challenged by transcription (Fig. 6).

## Maintenance pathways are active at non-imprinted genomic regions

In addition to the imprinted DMRs, a number of other methylated genomic sites are also targeted by Zfp57 and the ATRX/Daxx complex [12, 34, 69]. This includes the IAP retrotransposons which are heavily methylated and continuously protected from demethylation, even in primordial germ cells [15, 16]. In addition, both Zfp57 and ATRX/Daxx were found to localise with other heterochromatin modified regions including the 3′ end of zinc finger genes [12, 34]. Both IAP retrotransposons and the GC-rich 3′ intragenic zinc finger genes are also silenced by the KAP1 co-repressor complex [79, 80]. At IAP elements, KAP1 recruitment is predominantly dependent on Zfp809 [79]; however, Zfp57 and the ATRX/Daxx complex may provide an additional mode of recruitment or further stabilise the KAP1 interaction at IAPs. Given that aberrant expression of IAPs is deleterious to the genome [81], multiple redundant mechanisms for silencing would be important for ensuring genome integrity.

Similar to IAPs and imprinted methylated DMRs, the 3′ end of zinc finger genes are also enriched for heterochromatin modifications [82, 83]. This intragenic GC-rich regions may have the capacity to act as promoters and heterochromatin modifications would be required to prevent aberrant transcriptional initiation from these intragenic sites, not unlike the imprinted DMRs. As such, a highly similar complement of chromatin modifications and heterochromatin proteins are observed at these two classes of genes. This includes a combination of H3K36me3, H3K9me3 and DNA methylation which is linked to transcription and the recruitment of KAP1 respectively [8, 9, 80, 82]. In addition, the 3′ end of zinc finger genes are also enriched for HP1 and Zfp57 which presumably recruits KAP1 to these sites, as well as the ATRX/Daxx/H3.3 complex which can reinforce heterochromatin modifications throughout the cell cycle [12, 34]. The pathways for maintaining genomic imprinting are therefore also required for preserving heterochromatin at other non-imprinted genomic regions which nonetheless share features with the imprinted DMRs.

## Summary

The phenomenon of genomic imprinting is therefore dependent on the acquisition of differential methylation in parental germlines followed by the selective maintenance of imprints through development. Sex-specific differences during germline specification results in a differential pattern of DNA methylation in parental genomes at fertilisation. These differentially methylated regions include the imprinted gDMRs but are not exclusive to these sites. The differential methylation at non-imprinted regions is subsequently erased during the pre-implantation period when active and passive demethylation processes occur indiscriminately across the genome, except at regions which are specifically protected.

The maternal genome and paternally methylated gDMRs are protected from active Tet3 mediated demethylation via H3K9me2 dependent recruitment of PGC7. Silencing modifications at gDMRs are then further reinforced by the binding of Zfp57 and the KAP1 co-repressor complex which recruits both DNMT3a/b and Setdb1 to catalyse DNA methylation and H3K9me3 respectively. The continuous protection of silencing modifications at gDMRs is further facilitated by the ATRX/Daxx complex which deposits the replication independent H3.3 histone variant. Daxx reportedly interacts with KAP1 and Setdb1 which modifies H3.3 to H3.3K9me3, thus ensuring the faithful protection of heterochromatin modifications at gDMRs, even when subjected to transcription. In addition to the methylated gDMRs, both Zfp57 and the ATRX/Daxx/H3.3 complex also bind other methylated regions such as IAP retrotransposons and the 3′ end of zinc finger genes. Thus, neither the acquisition of differential methylation nor the maintenance processes are exclusive to imprinted gDMRs, and neither process is deterministic for genomic imprinting. Instead, the imprinted gDMRs arise from the intersection of these two pathways and it is this combinatorial interaction which ultimately distinguishes imprinted gDMRS from the rest of the genome.
